# Is Greenness Associated with Dementia? A Systematic Review and Dose–Response Meta-analysis

**DOI:** 10.1007/s40572-022-00365-5

**Published:** 2022-07-20

**Authors:** Federico Zagnoli, Tommaso Filippini, Marcia P. Jimenez, Lauren A. Wise, Elizabeth E. Hatch, Marco Vinceti

**Affiliations:** 1 Environmental, Genetic and Nutritional Epidemiology Research Center (CREAGEN), Department of Biomedical, Metabolic and Neural Sciences, University of Modena and Reggio Emilia Medical School, 287 Via Campi, 41125 Modena, Italy; 2 School of Public Health, University of California Berkeley, Berkeley, CA 94704 USA; 3 Department of Epidemiology, Boston University School of Public Health, Boston, MA 02118 USA

**Keywords:** Greenness, Green space, Dementia, Cognitive impairment, NDVI, Land use

## Abstract

**Purpose of Review:**

We assessed the relation between environmental greenness and risk of dementia and cognitive impairment, based on a systematic review and meta-analysis up to March 30, 2022, characterizing whenever possible the shape of the association using dose–response meta-analysis.

**Recent Findings:**

Twelve studies were included in this review, either using normalized difference vegetation index (NDVI) or land use/cover (LU/LC) methodology to assess greenness. Comparing the highest versus lowest exposure categories of greenness assessed using the NDVI (6 studies) or LU/LC (6 studies), we found no association with dementia. Dose–response meta-analysis of the association between greenness measured by LU/LC and dementia, based on only 3 studies, indicated a U-shaped association, but estimates were imprecise.

**Summary:**

Our systematic review and meta-analysis provided some evidence of a slight inverse association between greenness and dementia at intermediate exposure levels, but not at high levels. Potential methodological limitations, such as exposure misclassification and unmeasured confounding, may have affected the results.

**Supplementary Information:**

The online version contains supplementary material available at 10.1007/s40572-022-00365-5.

## Introduction

Dementia is a progressive neurodegenerative disease that results in major economic and social costs for both patients and their families. Approximately 50 million people in the world currently suffer from dementia; the estimated number of annual incident cases is 10 million, and approximately 150 million prevalent cases are predicted in 2050 [1]. For these reasons, dementia prevention is a key public health priority [2••]. Cognitive impairment is a clinical state that precedes dementia [3]. It is characterized by lower-than-expected performance in one or more cognitive domains considering the patient’s age and educational attainment; however, it does not substantially interfere in activities of daily living (ADLs). On the contrary, in dementia, more than one cognitive domain is affected, resulting in significant interference in ADL [3–6].

The causes of dementia remain unknown. Many potential risk factors have been investigated, such as reduced physical activity [7], poor diet [8], comorbidities such as diabetes, hypertension, and cardiovascular disease [9–11], and other environmental, clinical, and genetic factors. Environmental factors in particular, including exposure to air pollution, heavy metals, and metalloids [12], are increasingly considered as possible risk factors [13].

To date, there are no effective interventions to prevent, delay, or treat cognitive impairment and dementia, with the exception of preventive measures for vascular dementia [14, 15]. Therefore, the identification of environmental factors as risk factors for dementia could help prevent the disease, or its related disorders, either of the Alzheimer’s type or belonging to other clinical forms. Among the possible risk factors for dementia is the reduced availability of residential green space (“greenness”). Natural vegetation or green space/greenness surrounding residential and workplaces has been hypothesized to be protective towards dementia [16]. Potential beneficial effects of greenness on brain structure and function include greater cortical thickness [17, 18••, 19] and reduced ventricular size [20]. Research has shown a protective effect of residential greenness for a number of other outcomes including all-cause mortality, obesity, cardiovascular and respiratory diseases, poor sleep quality [21••, 22–29, 30••], pediatric anxiety and depression, and adverse birth outcomes [31, 32, 33••, 34•, 35].

The exposure indices most frequently used in literature to measure greenness are normalized difference vegetation index (NDVI) and land use and land cover (LU/LC). NDVI is obtained by satellite imagery and uses the characteristic of chlorophyll in leaves to absorb visible light for photosynthesis and reflect light near the infrared. NDVI is computed as the ratio of the difference between the near-infrared region and red reflectance to the sum of these two measures [36]. NDVI ranges from − 1 to 1, with more positive values representing higher greenness level, and negative values representing bodies of water [21••]. Other studies have instead relied on land use (LU), an indicator of land cover types, and land cover (LC), reflecting the physical and biological surface of land [37]. Studies based on LU/LC employed a land cover dataset to determine the percentage of land use around a predetermined buffer or area around the participant’s address, with specific reference to the presence of parks or urban green, forests, or crops/cultivations [38, 39]. Rarely used indices for exposure assessment include the enhanced vegetation index, based on land surface reflectance of light spectrum as NDVI but including adjustment for tree canopy background [40], and the vegetation continuous fields, relying on the percentage of land covered by tree canopies [41].

Given the growing number of studies assessing the relation between greenness and both cognitive impairment and dementia and the recent availability of new statistical tools for the assessment of non-linear relations [42, 43], we performed a systematic review of the literature and a dose–response meta-analysis to characterize the association between residential greenness and cognitive outcomes.

## Methods

### Study Identification

In this review, we applied the guidelines of Preferred Reporting Items for Systematic Reviews and Meta-Analysis (PRISMA) statement [44, 45]. We performed a literature online search in the PubMed/MEDLINE and Embase databases until March 30, 2022 with no time restriction. Two authors (FZ and TF) independently screened publication titles and abstracts and evaluated full text for inclusion in the review. In case of conflicting evaluation, a third author (MV) was sought to resolve the disagreement.

According to the PECOS (Population, Exposure, Comparator, Outcome and Study design) guidelines [46], the specific research question is “In the adult population, what is the effect of greenness on risk of cognitive impairment or dementia from epidemiological studies?”.

Based on the research question, we selected epidemiological studies investigating exposure to greenness among adults (≥ 18 years old). We included research articles and not reviews, letters, or conference abstracts. We used search terms related to greenness, e.g., “green space,” “recreational parks,” “urban park,” “urban green,” and “vegetation,” as well as terms related to greenness measurement methods, i.e., “normalized difference vegetation index,” “land use,” “land cover,” and “vegetation index.” To investigate the outcome, we used terms related to dementia and cognitive impairment using MeSH terms and Explode terms for PubMed and Embase research, respectively. Only studies quantifying the prevalence, incidence, or mortality rate for dementia or cognitive impairment were considered, provided they used the International Classification of Disease (ICD), prescription of anti-dementia medication, hospital using scales for cognitive impairment diagnosis like Mini Mental State Examination (MMSE), and with dementia diagnosis during hospitalization. We included all types of observational studies with individual level data, i.e., cohort, case–control, cross-sectional, and ecological studies. From the original literature search, we excluded studies not reporting the aforementioned information. Supplemental Table S1 includes the details of our search strategies. No language restrictions were applied. If multiple studies used the same population, we considered the most recent report, which included the one the largest population, or the one specifically aimed at assessing dementia as outcome.

### Data Extraction

From each selected study, we extracted location, study design, total study population, number of cases, and number of controls if applicable, period of observation or year when the study data were collected, greenness evaluation methodology, type of outcome and its assessment method, and risk estimates with 95% confidence intervals (CI) taken from the fully adjusted model. We also extracted, whenever possible, all the details about the magnitude of greenness for each exposure category in which the study population was divided. When greenness was assessed using different areas or buffer diameters, we extracted data from the smallest area or buffer. We contacted the authors of the four publications in which detailed data were not provided [47–50] and obtained additional data needed to perform the analysis.

### Risk of bias assessment

We assessed the risk of bias (RoB) of the included studies using the Risk of Bias in Non-Randomized Studies of Exposure (ROBINS-E) tool [51, 52]. Two investigators (FZ and TF) assessed the risk of bias. Any discrepancy was resolved by MV. Supplemental Table S2 reports criteria for RoB evaluation. Studies were considered “low RoB” if all domains were rated at low risk; they were considered “moderate” or “high” RoB if one or more domains was at moderate or high RoB, respectively.

### Data Analysis

We performed a meta-analysis comparing the highest versus the lowest greenness categories or continuous increase of greenness (1 standard deviation or interquartile range or 0.1 unit or 10% increments) using a restricted maximum likelihood random effects model with inverse–variance estimation method. We also explored the shape of the association between greenness and risk of cognitive impairment or dementia through a dose–response meta-analysis using a one-stage approach in order to explore also non-linear association [53] as previously implemented in other fields [54–56]. For this purpose, we also extracted the mean value of greenness for each exposure category. If the mean was not available, we used the median or the midpoint of each exposure stratum. In case of highest and lowest open-ended exposure categories, we used a value 20% higher or lower than the boundary value, based on the ranges observed in studies with complete data [57, 58]. Studies that provided risk estimates only for continuous exposure were excluded from the dose–response analysis. We used a restricted cubic spline model with three knots at fixed percentiles (10th, 50th, and 90th) of greenness through the restricted maximum likelihood random effects model [59]. We provided a graphical overlay of study-specific trends using predicted curves showing the influence of variation across studies. Finally, we assessed the heterogeneity of included studies using the *I*^2^ statistics [60]. We used Stata software (v17.0, Stata Corp. College Station, TX, 2021) to perform all data analysis.

## Results

### Study Selection

**Fig. 1:**
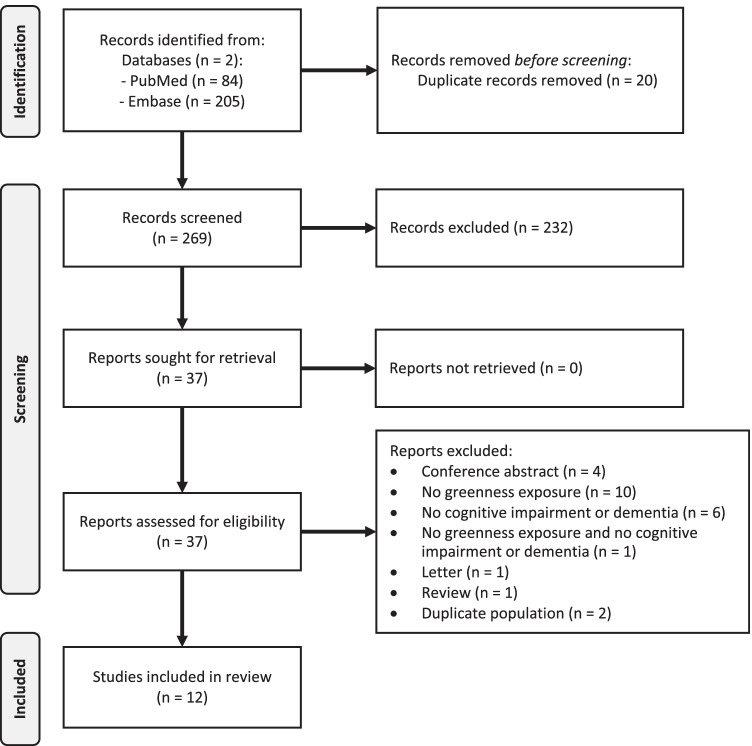
Flow diagram of study selection process

Figure 1 shows the PRISMA flow chart for literature search and study identification. We identified 269 potentially eligible articles, after the exclusion of duplicates. After analyzing the title and abstract, we discarded 232 papers and we retrieved the full text of the remaining 37. After their in-depth evaluation, we further excluded two studies because they analyzed the same population [61, 62••], 17 studies because they did not evaluate the exposure or outcome of interest (such as the two measuring cognitive decline but not cognitive impairment [19, 40]), and six papers because they were not original research articles. Overall, 12 publications eventually met the inclusion criteria, i.e., they had individual-level data and reported the association of greenness with dementia and/or cognitive impairment [47–50, 63–65, 66••, 67, 68••, 69, 70].

### Study Characteristics

Table 1 summarizes the main characteristics of included studies. The publication period ranged from 2015 to 2022. Three studies were conducted in Europe (two in UK [48, 67], one in the Netherlands [47]), five in North America (two in Canada [49, 66••] and three in US [68••, 69, 70]), three in Asia (one each in China [50], Taiwan [65], and Hong Kong [64]), and one in Australia [63]. Four studies had a cross-sectional design [48, 64, 67, 68••], one case–control [65], five cohort [47, 49, 63, 66••, 69], one including both cross-sectional and cohort design [50], and one ecological [70].

**Table 1:** Main characteristics of the selected studies

Reference	Study period	Country	Study design	Population	Exposure type	Area/buffer of exposure	Exposure distribution	Outcome	Measure of outcome	Adjustment variables
Aitken et al. 2021 [68••]	2011	Florida, USA	Cross-sectional	249,405 Medicare beneficiaries ≥ 65 years from US CMS	NDVI (ASTER 15 m)	Census Block	NDVI: mean − 0.02 (SD: 0.09); tertiles (ranges): T1 − 0.4, − 0.06; T2 − 0.06, 0.006; T3 0.006, 0.429	ADRD, AD, NAD	ICD-9 (CMS’ 2011 Chronic Conditions Segment)	Age, sex, race/ethnicity, neighborhood level income, comorbid health conditions
Astell-Burt et al. 2020 [63]	2006–2020 (11-year follow up)	New South Wales, Australia	Cohort	109,688 ≥ 45 years from Department of Human Services	LU/LC: Total green space, tree canopy, open grass (Landcover Data Pitney Bowes Ltd 2 m)	1.6 km of the point of residence	Total green space (range of categories): 0–24.9%, 25.0–31.9%, 32.0–39.9%, 40.0–49.9%, ≥ 50% Tree canopy: 0–9.9%, 10.0–19.9%, 20.0–29.9%, ≥ 30.0% Open grass: 0–4.9%, 5.0–9.9%, 10.0–19.9%, ≥ 20.0%	Dementia incidence	First anti-dementia medication (PBS); first hospitalization or death with dementia reported (ICD-10)	Sex, age, educational qualification, annual household income, couple status, area-level SE disadvantage
Ho et al. 2020 [64]	2007–2014	Hong Kong	Cross-sectional	228,600 ≥ 65 years from Hong Kong Census and Statistics Department mortality dataset	NDVI (IKONOS 15 m)	TPU of residence	NDVI: range − 0.37, 0.69; mean − 0.05 (0.1-unit increase in the analysis)	Dementia mortality	ICD-10: F00–F03 (Hong Kong Census and Statistics Department’s mortality dataset)	Age, sex, marital status, economically inactive, education, language, humidity, urban sky view, daily O_3_, NO_x_, PM_10_, day with low or high temperature, air pollution
Klompmaker et al. 2020 [47]	2013–2017	Netherland	Cohort	full (imputed) population: 339,633; complete case population: 244,814. Older than 30 years from PHM and Dutch longitudinal mortality database	NDVI (Landsat 5 30 m) and LU (TOP10NL)	300 m and 1000 m radius surrounding residential address	NDVI: median 0.52 (IQR 0.13); quintiles: Q1 ≤ 0.44, Q2 0.45–0.50, Q3 0.51–0.55, Q4 0.56–0.61, Q5 ≥ 0.62) LU: median 0.19 (IQR 0.24) (quintiles: Q1 ≤ 0.08, Q2 0.09–0.15, Q3 0.16–0.24, Q4 0.25–0.38, Q5 ≥ 0.39)	Neurodegenerative disease mortality and dementia mortality	ICD-10: F00–F03, G12.2, G20–22, G30, G35 (Statistics Netherlands)	Age, sex, marital status, region of origin, education, standardized household income, paid occupation, physical activity, BMI, smoking status, cigarettes smoked, alcohol status, glasses alcohol, PC4 composite SES, mean income neighborhood, unemployment neighborhood, percentage of immigrants’ neighborhood, mean income region, unemployment region, percentage of immigrants’ region
Liu et al. 2019 [65]	2010–2011	Taiwan	Case–control	24,802 older than 65 years (12,401 case, 12,401 control) from Taiwan’s NHIRD	LU (2006 National LU Investigation)	residential township	Environmental features (parks, greeneries and square area): ranges 0–26; mean 0.54 (SD 1.50, IQR 0.41) (IQR increase in the analysis)	AD	3 outpatient claims with AD ICD-9 and first and last visits in study period	Physical environment factors, social environment factors, occupational status, number of comorbidities, insurance premium, urbanization and hospitals and clinics status
Paul et al. 2020 [66••]	2001–2013	Ontario, Canada	Cohort retrospective	dementia cohort: 1,737,460 aged 55–85 years from ONPHEC	NDVI (Landsat 5 30 m)	250 m and 500 m buffer size from centroid city block	NDVI range 0, 0.7; mean 0.41 (IQR 0.12) (IQR increase in the analysis)	Dementia incidence	First anti-dementia medication: first hospitalization with dementia reported; 3 physician claims (ICD-9)	Age, sex, SES, comorbidities (coronary heart disease, diabetes, hypertension, congestive heart failure, stroke, arrhythmia) population density, air pollution, northern residence, family physician density, Deprivation Index, neurologist density
Slawsky et al. 2022 [69]	2000–2008	North Carolina, Maryland, Pennsylvania, California USA	Cohort	3047 older than 75 years from GEMS	NDVI (MODIS)	2000 m radius surrounding residential address	NDVI: mean 0.57 (SD: 0.13); tertiles (mean): T1 0.46 (SD 0.11); T2 0.57 (SD 0.08); T3 0.68 (SD 0.09)	Incidence of dementia, AD and VaD	NINCDS-ADRDA and a combination of CDR, ADAS and 3MS	Age, sex, ethnicity, BMI, smoking status, alcohol consumption, ADL, APOE ε4 genotype, recruitment site, neighborhood socioeconomic status, education, mild cognitive impairment at baseline, rurality
Wu et al. 2015 [48]	2001	UK	Cross-sectional	2424 older than 65 years old from CFAS	LU (Generalized LU 2001 dataset)	LSOA	LU mix: quartiles: Q1 0.06–0.45, Q2 0.46–0.64, Q3 0.65–0.72, Q4 0.73–0.88 Natural environment: quartiles: Q1 11.6–57.2%, Q2 57.3–65.7%, Q3 65.8–86.6%, Q4 86.7–98.5%	Incidence of cognitive impairment and dementia	MMSE e GMS	Age, sex, education, social class, number of chronic illnesses, area deprivation
Wu et al. 2017 [67]	2008–2011	England, UK	Cross-sectional	7505 older than 65 years from CFAS II	LU mix and LU natural environment (Generalized LU 2005)	LSOA	LU mix: quintiles: Q1 0.08–0.23, Q2 0.24–0.54, Q3 0.55–0.64, Q4 0.65–0.73, Q5 0.73–0.90 Natural environment: quintiles: Q1 9.5–55.6%, Q2 55.7–63.2%, Q3 63.3–74.7%, Q4 75.4–93.6%, Q5 93.7–98.0%	Incidence of cognitive impairment and dementia	MMSE e GMS	Age, sex, education, numbers of chronic illness, area deprivation
Wu et al. 2021 [70]	2011–2013	Mid-Atlantic USA	Ecological study	Mid-Atlantic US population older than 65 years with Medicare assistance	LC (Cheapeake Bay Watershed Land Cover 2013/2014)	ZIP code	LU: range 7–100%; mean 85% (SD 18%) (10% increase in the analysis)	AD	ICD-9	Population density, median income, waterbody presence, PM_2.5_
Yuchi et al. 2020 [49]	exposure period 1994–1998; follow-up period 1999–2003	Metro Vancouver, Canada	Cohort and case/cohort	4 cohorts 45–85 years: 633,949 NAD 634,432 PD 13,498 AD 7232 MS	NDVI (Landsat ETM +)	100 m radius surrounding residential address	NDVI: NAD: range − 0.1, 0.6; median 0.2 (IQR 0.11) AD: range 0, 0.6; median 0.3 (IQR 0.12) (IQR increase in the analysis)	Incidence of AD, NAD, PD, MS	NAD and AD: first anti-dementia medication; first hospitalization with dementia reported; 3 physician claims. PD: 2 physician claims, prescription. MS: first hospitalization with MS. Diagnosis by ICD 9/10 during hospitalization	Age, sex, comorbidities, household income, education, ethnicity
Zhu et al. 2019 [50]	2000–2014	China	Cross-sectional and cohort	Cross-sectional: 38,327; cohort: 19,726. Older than 65 years from CLHLS	NDVI (MODIS by Terra Satellite)	500 m radius surrounding residential address	NDVI; mean 0.40 (SD 0.15); quartiles: Q1 − 0.09, 0.30; Q2 0.30–0.44; Q3 0.44–0.51; Q4 0.51, 0.76	Cognitive impairment	MMSE (Chinese version with 24 self-reported questions)	Age, sex, ethnicity, marital status, urban/rural residence, education, occupation, financial support, social and leisure activity, smoking status, alcohol consumption, physical activity. (Number of years for each follow-up survey since entering the cohort)

*3MS* Modified Mini-Mental State Examination, *AD* Alzheimer’s dementia, *ADAS* Alzheimer’s Disease Assessment Scale, *ADL* activity of daily life, *ADRD* Alzheimer’s disease and related dementia; *ALFA* ALzheimer and FAmilies, *ASTER* Advanced Spaceborne Thermal Emission and Reflection Radiometer, *BMI* body mass index, *APOE ε4* apolipoprotein E ε4, *CDR* Global Clinical Dementia Rating, *CFAS* Cognitive Function and Ageing Study, *CLHLS* Chinese Longitudinal Healthy Longevity Survey, *CMS* Center for Medicare and Medicaid Services, *GEMS* Ginkgo Evaluation of Memory Study, *GMS* geriatric mental status, *ICD* International Classification of Diseases, *IQR* interquartile range, *Landsat ETM* + Landsat Enhanced Thematic Mapper Plus, *LSOA* lower-layer super output area, *LU/LC* land use/land cover, *MMSE* Mini Mental State Examination, *MODIS* Moderate Resolution Imaging Spectroradiometer, *mPACC* modified Preclinical Alzheimer Cognitive Composite, *MS* multiple Sclerosis, *NAD* non-Alzheimer’s dementia, *NDVI* normalized difference vegetation index, *NHIRD* National Health Insurance Research Data, *NINCDS-ADRDA* National Institute of Neurological and Communicative Disorders and Stroke/Alzheimer’s Disease and Related Disorders Association, *PBS* Pharmaceutical Benefits Scheme, *PC4* four-digit postal code level, *PD* Parkinson disease, *PHM* Public Health Monitor, *ONPHEC* Ontario Population Health and Environment Cohort, *SD* standard deviation, *SE* socio-economical, *SES* socioeconomic status, *TPU* tertiary planning unit, *VaD* vascular dementia, *ZIP* zoning improvement plan

With regard to exposure assessment, six studies measured greenness using NDVI [49, 50, 64, 66••, 68••, 69], five used LU/LC with the corresponding datasets for the classification of green areas [48, 63, 65, 67, 70], and one study presented both LU/LC and NDVI [47]. Six studies compared the greenness of the smallest residential units of study participants based on the administrative subdivision of each country [48, 64, 65, 67, 68••, 70], defined by an average population (e.g., lower layer super output areas [48, 67]) or on natural or urban boundaries such as roads (e.g., census block [68••]). In the other studies, residential greenness was measured using the mean value of NDVI or LU/LC with various dimensions of buffer around the residential addresses: one study analyzed residential greenness around a 1.6-km area [63]; another measured greenness at 250-m and 500-m buffers from centroid city block of subject’s residence [66••]; one study assessed the areal greenness of 100 m surrounding residential address [49]. The area considered by another study is 500-m buffer size around participant’s residence [50]; one study analyzed the area of 300 m and 1000 m around the residence [47]; another study analyzed the greenness at 2000-m buffer around the residence [69]. For data analysis, we used the values related to the smallest buffers, in accordance with previous reviews [71, 72], that ranged from 100 m to 2 km across the different studies. The characteristics of exposure assessment are described in more detail in the Supplemental Table S3.

All the three studies of cognitive impairment were based on categorical definitions of greenness, while seven out of the 11 studies on dementia used continuous exposure data, and the remaining four used categorical data.

As shown in Table 1, two studies investigated the association between greenness and mortality from dementia [47, 64], while four studies explored the association with incidence [49, 63, 66••, 69], one with further subdivision into all-cause dementia, vascular dementia, and Alzheimer’s disease [69]. Similarly, one study evaluated the association between greenness and incidence of four neurodegenerative diseases, namely Parkinson’s disease, multiple sclerosis, Alzheimer’s disease, and non-Alzheimer’s dementia, although only the latter two were considered for the analysis [49]. In addition, two studies investigated the association between greenness and both cognitive impairment and dementia [48, 67], while one study assessed cognitive impairment specifically [50]. One study investigated the association with Alzheimer’s dementia using prevalent cases [65], while one study considered prevalent cases of “Alzheimer’s disease and related dementias” (ADRD) together and divided into “Alzheimer’s disease” (AD) and “non-Alzheimer’s dementia” (NAD) [68••]. One ecological study design [70] used Alzheimer’s disease rate by zip code.

Four studies used the International Classification of Disease, Injuries and Causes of Death (ICD versions 9 and 10) to measure outcome [47, 64, 68••, 70]. Another four studies used for the diagnosis of dementia, in addition to ICD, hospitalization and/or the first treatment with specific drugs and/or physician claims [49, 63, 65, 66••]. Three studies administered established cognitive tests (e.g., MMSE and geriatric mental status) [48, 50, 67], though one of them was based on an adapted version of the MMSE [50]. One study used a series of clinical and neuropsychological tests for diagnosis of dementia classified according to DSM-IV criteria [69].

Five studies included a study population of over 200,000 individuals [47, 49, 64, 66••, 68••], one between 100,000 and 200,000 [63], two between 10,000 and 100,000 [50, 65], and three studies analyzed a population less than 10,000 [48, 67, 69]. The total number of study participants included in this review was over 3,350,000, including over 275,000 and over 24,000 cases of dementia and cognitive impairment, respectively. The ecological study population comprised subjects aged > 65 years, who lived in the Mid-Atlantic USA and had Medicare insurance [70].

After assessing the risk of bias of the included studies (Supplemental Table S4), we evaluated five studies as being at low RoB [47, 48, 63, 67, 69]. Five studies had moderate RoB [49, 64, 65, 66••, 68••] due to lack of education data, lack of detailed information about the magnitude of greenness, or with more than 10% of the population excluded to missing data. Two studies had a high RoB: one analyzed the relation between LU/LC and self-reported cognitive impairment [50], and the second examined the relation between NDVI and dementia without controlling for age and sex [70]. We performed a sensitivity analysis excluding such studies with high RoB.

### Greenness and Dementia

**Fig. 2:**
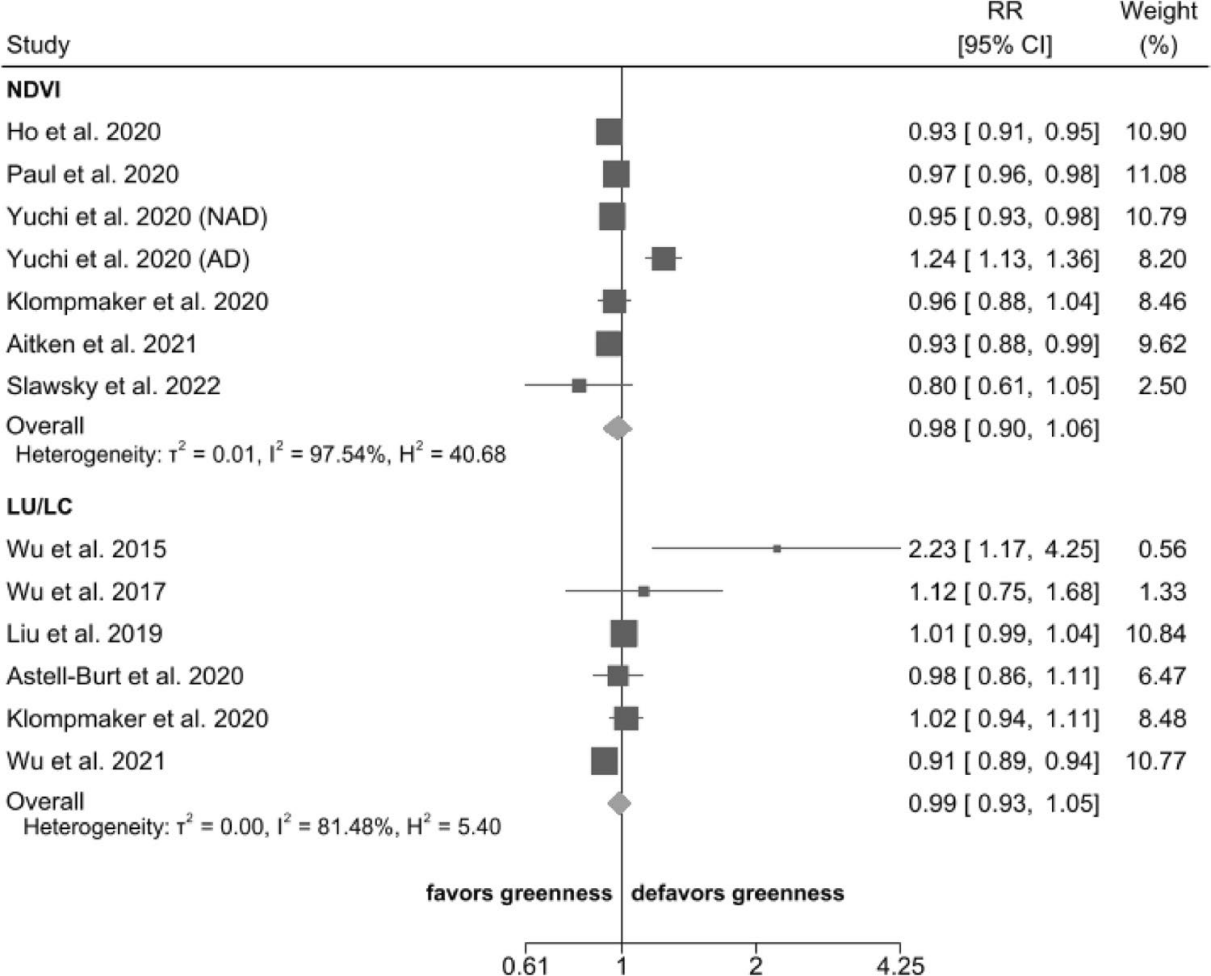
Risk ratio (RR) with 95% confidence interval (CI) between exposure to greenness measured by normalized difference vegetation index (NDVI) or land use/land cover (LU/LC) and dementia. The squares represent risk estimate, and horizontal lines represent their 95% CI. The area of each square is proportional with the weight of the study in the meta-analysis. The diamonds represent the combined risk for each type of exposure, and the solid line represents null value. The inverse-variance estimation method was used for study weighting. AD, Alzheimer’s disease; NAD, non-Alzheimer’s dementia

Figure 2 shows individual and summary risk ratios (RRs) of dementia comparing the highest to lowest greenness categories, or in case of continuous data for 1-unit exposure increase. We used a dose value 20% higher or lower of the boundary value in one study [63]. Overall, the summary RR for the association between greenness and dementia was 0.98 (95% CI 0.90–1.06) when greenness was measured with NDVI and 0.99 (95% CI 0.93–1.05) when LU/LC models were used. Stratification by types of study (e.g., longitudinal vs cross-sectional) and greenness measurement methodologies showed similar results (Supplemental Figure S1 and Supplemental Figure S2). The analysis restricted to Alzheimer’s disease as the outcome available in five studies yielded similar results: in the three studies [49, 68••, 69] using NDVI, the summary RR was 1.03 (95% CI 0.83–1.28), while in the two studies using LU/LC [65, 70], the summary RR was 0.96 (95% CI 0.87–1.06) (Supplemental Figure S3). Conversely, risk of non-Alzheimer’s dementia was reported in two studies only, one study [49] reporting a RR of 0.95 (95% CI 0.93–0.98) for interquartile increase of greenness and the second study [68••] reporting a RR of 0.95 (95% 0.89–1.02) in the intermediate exposure category and RR of 1.01 (95% CI 0.93–1.08) in the highest exposure group.

The association between dementia and greenness comparing the highest versus the lowest exposure categories, after excluding six studies based on 1-unit continuous exposure increase, showed a higher estimate compared with the overall result (Supplemental Figure S4). Similar results were also obtained after excluding the only study [70] at high risk of bias (Supplemental Figure S5).

**Fig. 3:**
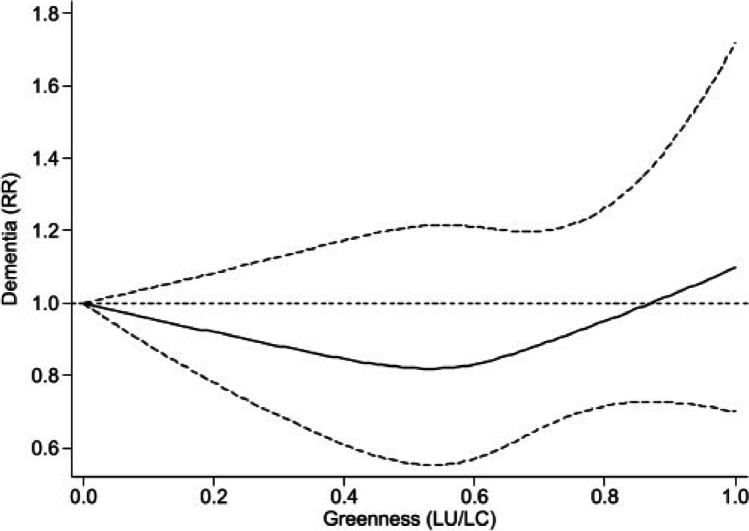
Dose–response of dementia and greenness measured by land use/land cover (LU/LC). Spline curve (black solid line) with 95% confidence limits (black dashed lines). RR, relative risk

The dose–response curve between dementia and greenness as measured by LU/LC showed a lower risk ratio for the “intermediate” range of exposure, between 0.2 and 0.8, with the lowest RR of around 0.8 at a greenness level of 0.5. At the highest levels of greenness, there was little evidence of protection, with the RR approaching and slightly exceeding 1 (Fig. 3). Two of the three included studies demonstrated a U-shaped relation while the other suggested no association [48, 63, 67] (Supplemental Figure S6). A dose–response meta-analysis between dementia and greenness based on the NDVI methodology could not be computed since only two studies were suitable for inclusion.

### Greenness and Cognitive Impairment

**Fig. 4:**
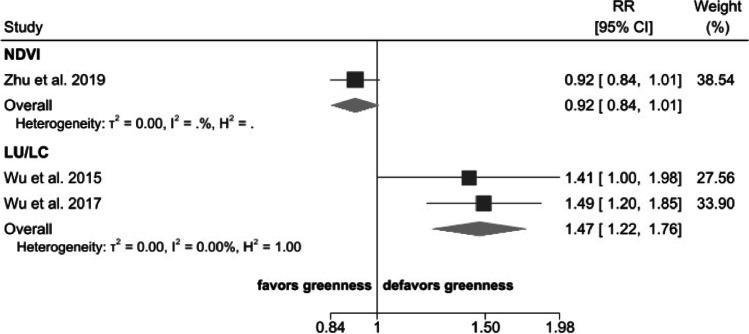
Risk ratio (RR) with 95% confidence interval (CI) between exposure to greenness measured by normalized difference vegetation index (NDVI) or land use/land cover (LU/LC) and cognitive impairment. The squares represent risk estimate, and horizontal lines represent their 95% CI. The area of each square is proportional with the weight of the study in the meta-analysis. The diamonds represent the combined risk for each type of exposure, and the solid line represents null value. The inverse–variance estimation method was used for study weighting

The two studies that investigated greenness using LU/LC and cognitive impairment reported a summary RR of 1.47 (95% CI 1.22–1.76) when comparing the highest and lowest exposure categories (Fig. 4). Only one study, having high RoB, assessed greenness with NDVI [50], and reported an odds ratio of 0.92 (95% CI 0.84–1.01).

We could not compute a dose–response meta-analysis between greenness and cognitive impairment due to the small number of studies available, whether using NDVI (*n* = 1) or LU/LC (*n* = 2).

## Discussion

Environmental greenness will have greater importance to humans given the progressive shift towards urbanization, with over 60% of the population projected to live in urban areas by 2030 [73]. The present systematic review and meta-analysis suggests that greenness may have a non-linear association with dementia. Specifically, the dose–response curve for greenness measured by LU/LC is consistent with the hypothesis that living in a place with an intermediate greenness value may protect against dementia. This U-shaped association may be related to possible counteracting effects and interplay of greenness-associated factors, both beneficial (reduced air pollution and noise annoyance, psychological factors, and increased physical activity) and adverse (greater social isolation, decreased interaction with neighbors, increased loneliness, and distance from medical and social services, particularly in rural areas) [16, 74–76]. However, such possible mediators and confounders were not adequately assessed in the available studies, and should be carefully considered in future investigations [77, 78]. The null findings from the categorical analysis do not necessarily contradict the dose–response analysis. Because the categories of exposure were defined independently in each study and were heterogeneous across studies, the pooled analysis is difficult to interpret and may be misleading. In addition, since the dose–response meta-analysis indicated comparable RRs in the highest and the lowest exposure ranges, the null results of the categorical analysis were not surprising as they could not capture the shape of the association between greenness and dementia at intermediate exposure levels.

NDVI and LU/LC were the greenness indices used in the papers that could be retrieved in this review. NDVI is an index obtained through satellite images [36] that cannot distinguish the type of “green,” while the LU/LC index, generated through regional databases, may better represent greenness typology. LU/LC has the additional advantage to reflect the presence of anthropogenic activities and related interactions with the natural ecosystem [38]. Therefore, while NDVI is an overall measure of green area, with no relation with greenspace use, LU/LC models may assess greenness as proximity of natural environments like forest and agricultural crops, owing to information generally yielded by regional datasets. Therefore, studies based on LU/LC might be able to provide more information than NDVI to investigate types of greenness. In addition, these studies may be affected by confounding, such as the different availability of facilities like recreational and social meeting places associated with proximity to green spaces, thus raising relevant methodological issues that have not been fully addressed in existing studies. These aspects may be particularly important in the study of dementia, since social isolation has been suggested as possible adverse effect of greenness when assessing its relation with cognition [77–79]. In this regard, LU/LC might be more valid than NDVI in providing an adequate characterization of green areas, accounting for these issues. Unfortunately, the number of studies based on LU/LC and addressing cognitive outcomes is limited.

The observational nature of included studies and the lack of detailed information for some potential mediators and/or confounders and their interactions represent major limitations of the studies on greenness and cognitive outcomes. In particular, it is difficult to identify the exact factors independently associated with greenness and that may mediate potential favorable effects on dementia, such as decreased stress levels [80, 81], lower air pollution [82•, 83, 84•], increased physical activity [23, 85], reductions in obesity [86], and improved mental health [87, 88].

Heterogeneity in the definition of greenness across studies, particularly in terms of area characteristics and size, could have hampered the detection of an association with cognitive impairment and dementia. For instance, to define greenness, some studies chose the buffer size area from residence [47, 49, 50, 63, 66••, 69], e.g., area of 1.6 km from the point of residence because it is the distance that a person can cover on foot and because long distances allow opportunities for contact with more green spaces [88]. In other studies [48, 64, 65, 67, 68••, 70], the area considered to assess greenness coincided with the smallest geographic unit used by the national demographic agency, e.g., the census block level NDVI for each study participants’ residential address in a Florida study [68••]. Similarly, the timing of exposure assessment differs across studies, since some of them assessed greenness at several time points during the follow-up [66••], or reported the average value of four seasons [50], or alternatively relied on a single measure, as done in most studies. In addition, timing of exposure assessment did not consider changes in greenness over time, before disease onset. Another source of exposure misclassification stems from the fact that greenness was generally measured surrounding the participants’ address of residence, while the time spent in other places (such as during working and recreational activities) was not taken into consideration. In addition, a source of heterogeneity across studies and of bias may have been the different confounders considered in the analyses. All studies except one [70] were adjusted for socio-economic status, which may be a relevant confounder [89], while only two studies [64, 66••] accounted for air pollution, also an important covariate. Air pollution has been associated with a large spectrum of neurological disorders including its ability to affect the incidence and mortality of dementia [90]. Considering the observation that green spaces, especially urban green spaces, may have the ability to reduce air pollutant levels [91], the latter can be both a mediator and a confounder [92–94] when addressing the role of greenness in the etiology of cognitive impairment and dementia. In the only study evaluating the association between greenness and dementia risk with and without air pollution in the multivariate model, the hazard ratio became weaker in the full adjusted model [66••].

With reference to outcome assessment, a potential source of heterogeneity was the different methodologies used across studies. For dementia diagnosis, some studies used ICD classification, while others used first anti-dementia medication, first hospitalization with dementia reported, and/or physicians’ diagnosis, or other tools such as Global Clinical Dementia Rating. In contrast, all studies used the MMSE for the diagnosis of cognitive impairment.

We acknowledge additional limitations of our review and the underlying studies on which it was based. The small number of available studies hampered our assessment, yielding statistically imprecise summary risk estimates and hampering the possibility to carry our subgroup analyses, including the exploration of potential effect modifiers such as sex, age, race, socioeconomic status, and the different associations with dementia types.

## Conclusions

This systematic review and meta-analysis suggests that environmental greenness may have a non-linear association with dementia. Specifically, the data are consistent with the hypothesis that living in a place with an intermediate greenness value may protect against dementia. Given the limitations of previous studies in terms of exposure assessment, control of confounding, and lack of precision, future studies should address these methodological challenges to facilitate pooled analyses and to provide more reliable conclusions.

## Supplementary Information

Below is the link to the electronic supplementary material.

Supplementary file1 (DOCX 648 kb)
