# Supplementary material for: Is Greenness Associated with Dementia? A Systematic Review and Dose–Response Meta-analysis
Source: Curr Environ Health Rep. 2022 Jul 20;9(4):574–90. doi: 10.1007/s40572-022-00365-5 (PMC9729322; doi:10.1007/s40572-022-00365-5)

**Supplemental Table S1**. Database search strategies.

| **DATABASE** | **SEARCH STRATEGY** |
| --- | --- |
| PubMed | ("Dementia"[MeSH Terms] OR "Alzheimer Disease"[MeSH Terms] OR ("parkinson disease, secondary"[MeSH Terms] OR "Parkinson Disease"[MeSH Terms] OR "Parkinsonian Disorders"[MeSH Terms]) OR ("Dementia"[MeSH Terms] OR "Dementia"[All Fields] OR "dementias"[All Fields] OR "dementia s"[All Fields]) OR "Neurocognitive Disorders"[MeSH Terms]) AND ("ndvi"[All Fields] OR "lulc"[All Fields] OR ("land use"[All Fields] OR "land cover"[All Fields]) OR "green index"[All Fields] OR ("greenness"[All Fields] OR "green space"[All Fields] OR "green spaces"[All Fields] OR "greenspace"[All Fields] OR "greenspaces"[All Fields] OR ("canopied"[All Fields] OR "canopies"[All Fields] OR "canopy"[All Fields]) OR "tree canopy"[All Fields] OR "parks, recreational"[MeSH Terms] OR ("urban park"[All Fields] OR "urban parks"[All Fields]) OR "urban tree"[All Fields] OR ("vegetation"[All Fields] OR "urban green"[All Fields]))) |
| Embase | ('greenness'/exp OR 'green space'/exp OR ndvi OR 'land use'/exp/mj OR 'urban green space'/exp OR 'recreational park'/exp) AND ('cognitive defect'/exp OR 'dementia'/exp) |

**Supplemental Table S2.** Criteria for Risk of Bias assessment.

| **DOMAINS** | **CRITERIA** |
| --- | --- |
| BIAS DUE TO CONFOUNDING | The factors considered fundamental to ensure that the study is at low risk of bias (RoB) are age and education. Study is considered at moderate RoB if considered only age as confounding factor. Study is considered at high RoB if adjusting factors are not reported. |
| BIAS IN SELECTION | Selection of study participants yielded appropriate comparison groups and they have good representative. The descriptions of the studied population were detailed to support the affirmation that risk of selection effects was minimal. If the selection of participants is not related to greenness, the study is considered to be at low RoB. Study is considered at high risk if the information of modality of selection is missing. |
| BIAS IN CLASSIFICATION OF EXPOSURES | Check that exposure to greenness was assessed prior to outcome and how it was evaluated. The study is considered at low RoB if the exposure is measured via satellites or using pre-established datasets. Study is considered at high RoB if it relied on self-report for exposure classification or criteria are not reported. |
| BIAS DUE TO DEVIATION FROM INTENDED EXPOSURES | Study is considered at low RoB if exposure dose was reported. Study is considered at moderate RoB if exposure level was reported but not its dose in each category. Study is considered at high RoB if greenness levels was not reported. |
| BIAS DUE TO MISSING DATA | Study is considered at low RoB if less than 10% of study population was excluded to missing data, moderate RoB if less than 20%, high RoB if more than 20% of participant was excluded to missing data. |
| BIAS IN MEASUREMENT OF OUTCOMES | Possible bias based on the modality of outcome assessment (ICD or other validated classification, medication, mortality, hospitalization). High risk if assessment based on self-report. The RoB is moderate if outcome assessment was based on self-report with external validation. The RoB is low if outcome was classified based on diagnosis standard criteria; |
| BIAS IN SELECTION OF REPORTED RESULTS | Study is considered at low RoB if it reported a publication of the protocol or data are available in a public and accessible dataset. Study is considered at moderate RoB if it presented outcome measures and analyses was outlined in the manuscript. Studies are considered at high risk of bias if no protocol was available and the outcome measures and analyses weren’t outlined. |

**Supplemental Table S3.** Detailed characteristics of greenness exposure.

| Reference | Main greenness exposure assessment | LU/LC database | Other types of greenness measurement | Data imagery and spatial resolution | Exposure measurement period | Area/buffer exposure |
| --- | --- | --- | --- | --- | --- | --- |
| Aitken et al. 2021 [68] | NDVI | / | / | ASTER satellite imagery, 15 m | March 2011 | Census Block of residential address |
| Astell-Burt et al. 2020 [63] | LU/LC: Total green space | Western Australian Planning Commission and Landcover data from Data Pitney Bowes Ltd | Tree Canopy and Open Grass | 2 m | 2009 | 1.6 km of the point of residence |
| Ho et al. 2020 [64] | NDVI | / | Urban compactness by sky view factor | IKONOS, 15 m | / | Tertiary planning unit (TPU) of residence |
| Klompmaker et al. 2020 [47] | NDVI and LC | TOP10NL (land use database of Netherland) | / | LANDSAT_5, 30 m | Summer 2010 | 300 m and 1000 m radius surrounding residential address |
| Liu et al. 2019 [65] | LU: parks, greeneries and square area | National Land Use investigation | Playgrounds and sport venues | / | 2006 | Residential township |
| Paul et al. 2020 [66] | NDVI | / | / | LANDSAT_5, 30 m | Average annual exposure during study period | 250 m and 500 m buffer size from centroid city block |
| Slawsky et al. 2022 [69] | NDVI | Moderate Resolution Imaging Spectroradiometer by Terra satellite | Distance to parks and parks percentage | / | 7-day composite summer values | 2000 m radius surrounding residential address |
| Wu et al. 2015 [48] | LU: natural environment | Generalized Land Use dataset | Land use mix and Area deprivation | / | 2001 | Lower-layer super output area (LSOA) |
| Wu et al. 2017 [67] | LU: natural environment | Generalized Land Use dataset | Land use mix | / | 2005 | Lower-layer super output area (LSOA) |
| Wu et al. 2021 [70] | LC: tree (included shrubs) and herbaceous | Cheapeake Bay Watershed Land Cover | / | US Department of Agriculture, 1 m | 2013 and 2014 | Zoning improvement plan (ZIP) code |
| Yuchi et al. 2020 [49] | NDVI | / | / | Landsat Enhanced Thematic Mapper Plus | Average annual exposure during study period using 1999-2002 data | 100 m radius surrounding residential address |
| Zhu et al. 2019 [50] | NDVI | / | / | Moderate Resolution Imaging Spectroradiometer by Terra satellite | Average pf January, April, July and October from 2000 to 2014 | 500 m radius surrounding residential address |

**Notes:** LC, Land Cover; LU, Land Use; LU/LC: Land Use/Land Cover; NDVI, Normalized Difference Vegetation Index.

**Supplemental Table S4.** Risk of Bias of selected studies.

| Reference | Bias due to confounding | Bias in selection | Bias in classification of exposure | Bias due to deviation from intended exposure | Bias due to missing data | Bias in measurement of outcome | Bias in selection of reported results | Overall RoB |
| --- | --- | --- | --- | --- | --- | --- | --- | --- |
| Astell-Burt 2020 [63] | low | low | low | Low | low | low | low | low |
| Aitken 2021 [68] | moderate | low | low | low | low | low | low | moderate |
| Ho 2020 [64] | moderate | low | low | moderate | low | low | low | moderate |
| Klompmaker 2020 [47] | low | low | low | low | low | low | low | low |
| Liu 2019[65] | low | low | low | moderate | low | low | low | moderate |
| Paul 2020 [66] | moderate | low | low | moderate | low | low | low | moderate |
| Slawsky 2022 [69] | low | low | low | low | low | low | low | low |
| Wu 2015 [48] | low | low | low | low^a^ | low | low | low | low |
| Wu 2017 [67] | low | low | low | low^a^ | low | low | low | low |
| Wu 2021 [70] | high | low | low | moderate | low | low | moderate | high |
| Yuchi 2020 [49] | low | low | low | moderate | low | low | low | moderate |
| Zhu 2019 [50] | low | low | low | low^a^ | moderate | high | low | high |

**Notes**: ^a^Data provided by author: we requested through email the values of greenness when the population distribution was quantitative (e.g. quartiles) and the greenness dose for each subdivision was missing.

**Supplemental Figure S1.** Risk ratio (RR) with 95% confidence interval (CI) between exposure to greenness measured by Normalized Difference Vegetation Index (NDVI) and dementia divided by study design. The squares represent risk estimate and horizontal lines represent their 95% CI. The area of each square is proportional with the weight of the study in the meta-analysis. The diamonds represent the combined risk for each type of exposure, and the solid line represents null value. The inverse-variance estimation method was used for study weighting. AD, Alzheimer’s disease; NAD, non-Alzheimer’s dementia.


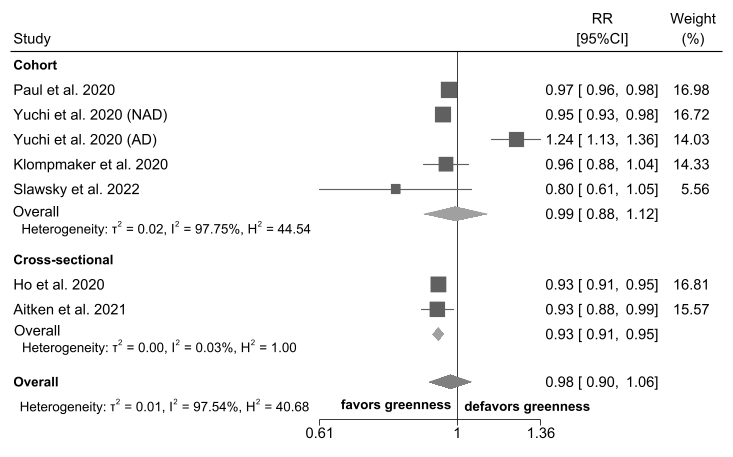


**Supplemental Figure S2.** Risk ratio (RR) with 95% confidence interval (CI) between exposure to greenness measured by Land Use/Land Cover (LU/LC) and dementia divided by study design. The squares represent risk estimate and horizontal lines represent their 95% CI. The area of each square is proportional with the weight of the study in the meta-analysis. The diamonds represent the combined risk for each type of exposure, and the solid line represents null value. The inverse-variance estimation method was used for study weighting.


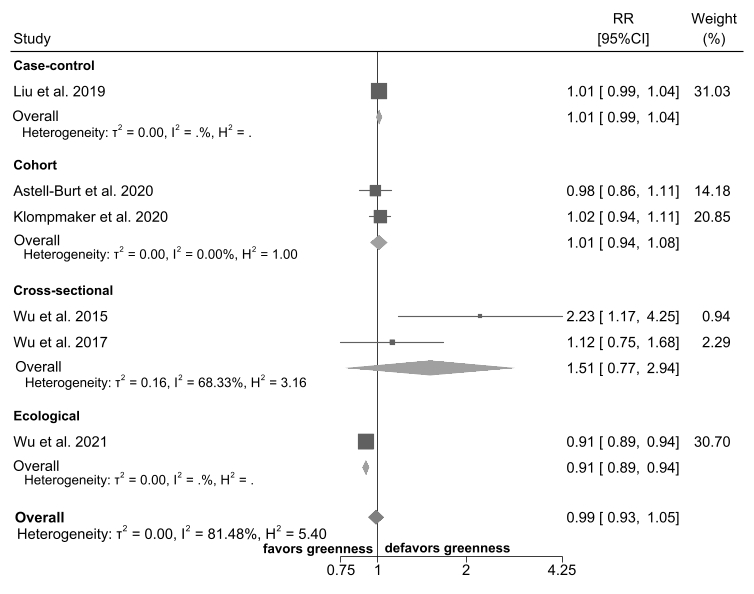


**Supplemental Figure S3.** Risk ratio (RR) with 95% confidence interval (CI) between exposure to greenness measured by Normalized Difference Vegetation Index (NDVI) or Land Use/Land Cover (LU/LC) and Alzheimer’s Disease. The squares represent risk estimate and horizontal lines represent their 95% CI. The area of each square is proportional with the weight of the study in the meta-analysis. The diamonds represent the combined risk for each type of exposure, and the solid line represents the null value. The inverse-variance estimation method was used for study weighting. AD, Alzheimer’s disease.


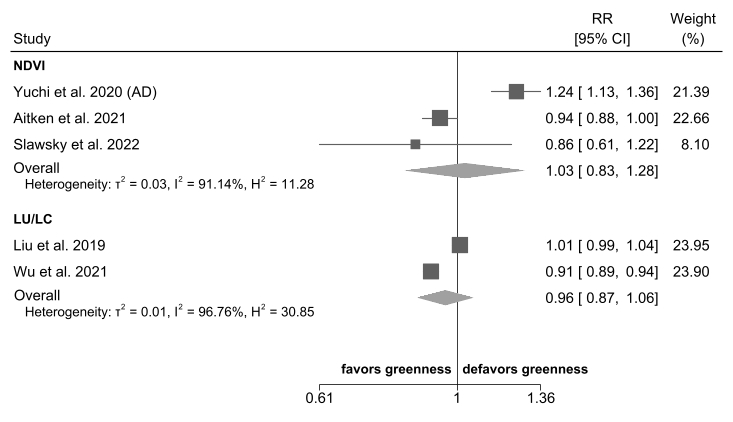


**Supplemental Figure S4.** Risk ratio (RR) with 95% confidence interval (CI) between exposure to greenness measured by Normalized Difference Vegetation Index (NDVI) or Land Use/Land Cover (LU/LC) and dementia comparing the highest versus the lowest exposure categories (excluding studies based on 1-unit continuous exposure increase). The squares represent risk estimate and horizontal lines represent their 95% CI. The area of each square is proportional with the weight of the study in the meta-analysis. The diamonds represent the combined risk for each type of exposure, and the solid line represents null value. The inverse-variance estimation method was used for study weighting.


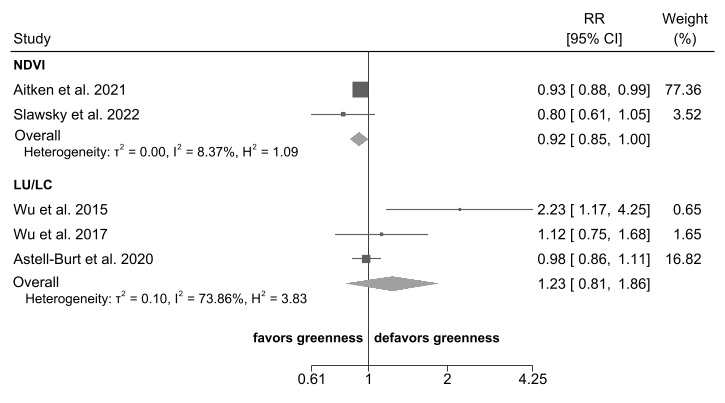


**Supplemental Figure S5.** Risk ratio (RR) with 95% confidence interval (CI) between exposure to greenness measured by Normalized Difference Vegetation Index (NDVI) or Land Use/Land Cover (LU/LC) and dementia, after excluding study with high risk of bias. The squares represent risk estimate and horizontal lines represent their 95% CI. The area of each square is proportional with the weight of the study in the meta-analysis. The diamonds represent the combined risk for each type of exposure, and the solid line represents null value. The inverse-variance estimation method was used for study weighting. AD, Alzheimer’s disease; NAD, non-Alzheimer’s dementia.


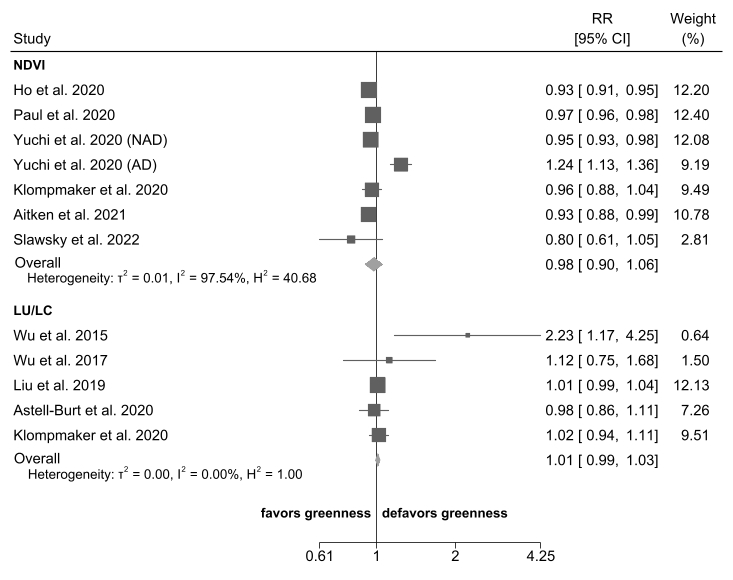


**Supplemental Figure S6.** Dose-response of dementia and greenness measured by Land Use/Land Cover (LU/LC). Spline curve (black solid line) with 95% confidence limits (black dashed lines). Trend of individual studies (grey lines). RR: relative risk.


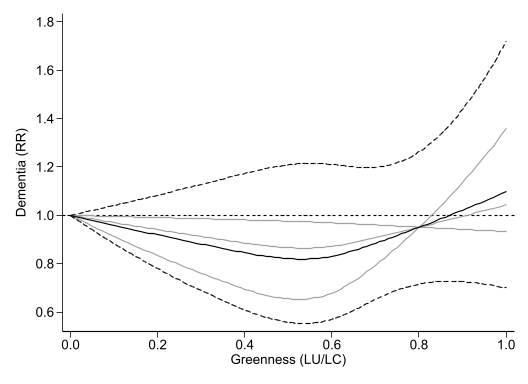

Supplement: Supplementary file 1 — Supplementary file1 (DOCX 648 kb) [file 40572_2022_365_MOESM1_ESM.docx]
